# Strains of the toxic and bloom-forming *Nodularia spumigena* (cyanobacteria) can degrade methylphosphonate and release methane

**DOI:** 10.1038/s41396-018-0056-6

**Published:** 2018-02-14

**Authors:** Jonna E. Teikari, David P. Fewer, Rashmi Shrestha, Shengwei Hou, Niina Leikoski, Minna Mäkelä, Asko Simojoki, Wolfgang R. Hess, Kaarina Sivonen

**Affiliations:** 1 Department of Microbiology, University of Helsinki, Viikinkaari 9, Helsinki, FI-00014 Finland; 2 Genetics and Experimental Bioinformatics, Institute of Biology III, University Freiburg, Schänzlestraße 1, Freiburg, D-79104 Germany; 3 Department of Agricultural Sciences, University of Helsinki, Viikinkaari 9, Helsinki, FI-00014 Finland

## Abstract

*Nodularia spumigena* is a nitrogen-fixing cyanobacterium that forms toxic blooms in the Baltic Sea each summer and the availability of phosphorous is an important factor limiting the formation of these blooms. Bioinformatic analysis identified a phosphonate degrading (*phn*) gene cluster in the genome of *N. spumigena* suggesting that this bacterium may use phosphonates as a phosphorus source. Our results show that strains of *N. spumigena* could grow in medium containing methylphosphonic acid (MPn) as the sole source of phosphorous and released methane when growing in medium containing MPn. We analyzed the total transcriptomes of *N. spumigena* UHCC 0039 grown using MPn and compared them with cultures growing in P_i_-replete medium. The *phnJ*, phosphonate lyase gene, was upregulated when MPn was the sole source of phosphorus, suggesting that the expression of this gene could be used to indicate the presence of bioavailable phosphonates. Otherwise, growth on MPn resulted in only a minor reconstruction of the transcriptome and enabled good growth. However, *N. spumigena* strains were not able to utilize any of the anthropogenic phosphonates tested. The phosphonate utilizing pathway may offer *N. spumigena* a competitive advantage in the P_i_-limited cyanobacterial blooms of the Baltic Sea.

## Introduction

Phosphorus is an essential macronutrient for life, being a key component in organic biomolecules, such as DNA, proteins, and phospholipids. The most preferable form of phosphorus for the uptake by cyanobacteria is orthophosphate ions H_2_PO_4_^2−^, HPO_4_^2−^, and PO_4_^3−^ (P_i_), which occur at an oxidation state of +5 in nature and these orthophosphates dominate the pool of dissolved inorganic phosphorus (DIP) [1]. Dissolved organic phosphorus (DOP) comprises another pool of phosphorus in the water ecosystems and includes two important bond classes, ester (C-O-P) and carbon-phosphorus (C-P) bonds. Phosphoesters are degraded by alkaline phosphatase and measurement of alkaline phosphatase activity has been used generally as an indicator for P_i_ deficiency [2, 3] (Van Wambeke et al. 2002). Organic phosphonates, derivatives of phosphorus acid where the phosphorus is at the oxidation state of +3, are poorly studied even though they have proposed to constitute up to 25 % of the total DOP pool in the oceans [4–6]. Many of the phosphonates in the DOP pool are natural metabolites but some have an anthropogenic origin [7–9]. Phosphonates are recalcitrant to degradation, due to the presence of the C-P bond, and are generally thought to particulate and sediment [1].

P_i_ is usually found at very low concentrations in environment and therefore lack of P_i_ is the main growth-limiting factor for nitrogen-fixing and phototrophic cyanobacteria during blooms in aquatic ecosystems [10–12]. Bacteria have evolved specific strategies to enhance phosphorus availability under P_i_-limited conditions [13, 14]. The high-affinity phosphate transport system, encoded in the *pstABCS* operon, is the most studied system for P_i_ uptake. The *pstABCS* operon belongs to the *pho* regulon, which is activated by autophosphorylation when the P_i_ concentration is low [15–17]. The PstABCS complex thus ensures rapid and effective scavenging of P_i_ in phosphorus-limiting conditions.

Many heterotrophic bacteria possess a phosphonate degrading (*phn*) gene cluster required for transport and assimilation of phosphonates that enables them to cope better with DIP-limited conditions by allowing them to use phosphonates as a source of phosphorus [18, 19]. The *phn* gene cluster is also part of the *pho* regulon and it consists of a phosphonate transporter complex (*phnC-E*) and the multi-subunit C-P lyase complex (*phnG-P*), which cleaves the C-P bond in phosphonates [20–22]. The PhnN, PhnO, and PhnP proteins are not required for C-P bond cleavage but they most probably have a role as accessory proteins or regulators [23]. The PhnF protein acted as a repressor of *phnC-E* in *Mycobacterium smegmatis* [24]. The cyanobacteria *Trichodesmium* IMS101, *Synechococcus* JA-2-3Ba(2–13), and *Anabaena cylindrica* PCC 7122 have been found to harbor full *phn* gene clusters including phosphonate transport and C-P lyase units, and can grow in medium containing phosphonates as a sole source of phosphorus [25–27]. These cyanobacteria contribute to methane supersaturation in the epipelagic zone of marine ecosystems through the degradation of MPn thus releasing methane into the surrounding environment [28, 29]. The MPn cycle may partially explain the oceanic methane paradox, where methane concentration in the surface waters is above the atmospheric equilibrium [4].

The diazotrophic cyanobacteria *Nodularia spumigena, Aphanizomenon spp*., and *Dolichospermum spp*. form annual toxic blooms in the Baltic Sea [30–32] despite low P_i_ concentrations (0.1–0.01 µM) [33, 34]. *N. spumigena* utilizes alternative phosphorus sources by degrading organophosphates using alkaline phosphatases [35]. However, the presence of a *phn* gene cluster in the genome of *N. spumigena* CCY 9414 suggested that this strain might be able to degrade and use phosphonates as an alternative source of phosphorous [36]. Supersaturation of methane has been detected in the surface waters of the Baltic Sea with great temporal variation [37]. Elevated methane concentration in the surface water was measured during the summer and early autumn coincidental with *N. spumigena* bloom formation [37, 38]. The aerobic release of the methane as a byproduct of MPn degradation could explain the reported peaks in methane concentration in the Baltic Sea [37, 38]. Here, we studied the capacity of axenic Baltic Sea *N. spumigena* strains isolated from the Baltic Sea to utilize phosphonates as the sole source of phosphorus and their ability to simultaneously release  methane. We analyzed the expression of phosphonate transporter (*phnD)*, phosphonate lyase (*phnJ*), and high-affinity phosphate transporter (*pstS*) genes of two Baltic Sea *N. spumigena* UHCC 0039 and 0060 strains and sequenced total transcriptomes of the cells growing in medium with MPn as a sole source of phosphorus and compared them with cells growing in the medium with P_i_. *N. spumigena* strains had the ability to degrade some phosphonates, which could represent an alternative source of phosphorus under P_i_-limiting conditions in the Baltic Sea. *N. spumigena* cyanobacteria released methane when MPn was present in the growth medium and the use of MPn as the sole source of phosphorus resulted in only a minor reconstruction of the transcriptome enabling good growth of *N. spumigena*.

## Materials and methods

### Screening for *phnJ* genes

The BlastP algorithm was used to identify PhnJ phosphonate lyase proteins from cyanobacterial genomes using the PhnJ sequences from *Escherichia coli* (*E. coli*) K12 and *N. spumigena* UHCC 0039 as queries. Genomes encoding the PhnJ protein were downloaded from the NCBI genome database (Table S1). The gene order of the *phn* gene cluster was determined using the Artemis genome browser [39]. A total of 16 strains of the genus *Nodularia* were selected for the screening for the occurrence and distribution of phosphonate lyase (*phnJ*) and transporter (*phnD*) genes (Table S2). *phnJ*, *phnD*, and *pstS* primers were designed based on the known *N. spumigena* UHCC 0039 (NCBI accession number, PRJNA352241), CCY 9414 sequences (NCBI accession number, PRJNA13447), and CENA 596 (NCBI accession number, PRJNA315832) (Table 1). *N. spumigena-*specific primers were also used to amplify *phnJ* and *phnD* genes from environmental DNA samples collected after cyanobacterial blooms in August 2016 (Table S3). Genomic DNA was isolated from the cyanobacterial cultures listed in Table S2 using the E.Z.N.A^®^ Plant DNA kit (Omega Biotek) and from environmental samples using AllPrep DNA/RNA mini kit (Qiagen). The PCR reaction mix (20 µL) consisted of 100 ng template genomic DNA, 750 nM of both primers (Oligomer, Table 1), 2 µl of 10× reaction buffer (Thermo Scientific), 200 µM dNTP (Thermo Scientific) and 0.4 U Dynazyme II (Thermo Scientific) in a total volume of 20 µL. Purified water was used as a template in negative controls. The PCR cycling parameters were as follows: 94 °C for 3 min, 30 × (94 °C for 30 s, 60.5 °C for 30 s, 72 °C for 180 s) and 72 °C for 10 min.

**Table 1:** *Nodularia spumigena* -specific primers used in this study

Target gene	Annotation	Primer	Sequence 5’– > 3'	Product size (bp)	Efficiency (%)	Melting temperature (°C)
phnD	Phosphonate transporter	phnDF	GGTGCCTGCGGATTCTGACA	225	98.8	63
		phnDR	TAACATCGCCGCGTCATGAG			60
phnJ	C-P bond lyase	phnJF	TTCTAGGGCGTGCATTTTGC	216	99.6	58
		phnjJR	ACCAACGCCGTGAATATTCG			58
pstS	Phosphate binding	pstSF	GTTGCAGCCAATGGCACT	119	99.3	56
		pstSR	CTTGACTTGTGCCAAACC			54
gyrB	Gyrase subunit B	gyrBF	CGCATATTCGCACACTGTTG	189	100.5	58
		gyrBR	TGTTGTAGTTGGCGTTGCTG			58

### Cyanobacterial strains and cultivation

*N. spumigena* strains UHCC 0039 (formerly named *N. spumigena* AV1) and UHCC 0060 (formerly named *N. spumigena* HEM) were isolated from the Gulf of Finland [32] and purified into axenic cultures (Table S1). The strains were maintained in continuous batch culture with Z8XS medium containing 17.1 mg L^–1^ of inorganic phosphate P_i_, without nitrogen and under continuous illumination of 3.2–3.7 μmol photons m^−2^ s^−1^ [40]. Cultures were starved in phosphorus-free medium (Z8XS-P) for 7 days to deplete the intracellular phosphorus store . Cells, in three biological replicates, were harvested after starvation and transferred to fresh Z8XS-P medium containing either methylphosphonic acid (MPn) (Sigma-Aldrich), ethylphosphonic acid (EPn) (Sigma-Aldrich), 2-aminoethylphosphonic acid (2Apn) (Sigma-Aldrich), etidronic acid monohydrate (Sigma-Aldrich), *N*-(phophonomethyl) glycine (Sigma-Aldrich), nitrilotri(methylphosphonic acid) (Sigma-Aldrich) or 2-phosphonobutane-1,2,4-tricarboxylic acid (abcr GmbH) as a source of phosphorus. The phosphorus concentration in each phosphonate medium was adjusted to be equal to the amount of phosphorus in the original Z8XS medium. Z8XS medium containing P_i_ was used as a positive control to gain proper cellular proliferation and Z8XS-P medium lacking P_i_ (-P_i_) was used as a negative control. Cultures were grown under continuous illumination of 3.2–3.7 μmol photons m^−2^ s^−1^ for 12 days. In all, 54 mL of the cultures were moved to 60 mL of fresh medium after 12 days of incubation and further cultured for 41/38 days. All glassware was acid washed using 0.1 M HCl.

### Determination of chlorophyll *a* concentration, alkaline phosphatase activity, and methane liberation

Chlorophyll *a* concentration and alkaline phosphatase activity were measured at 4-day intervals during the experiment. In all, 1 mL of culture was filtered through 21 mm glass microfiber filters GF/C (pore size 1.2 µm) (GE HealthCare) and stored in −80 °C for chlorophyll *a* measurements. Chlorophyll *a* was extracted from the filters using 1 mL of 90% acetone for 24 h at −20 °C and chlorophyll *a* concentrations were determined by measuring absorbance at 664, 647, and 630 nm. The chlorophyll *a* concentration was calculated using the Jeffrey and Humphrey [41] equation. Alkaline phosphatase activity was determined fluorometrically using 4-umbelliferyl phosphate as a substrate (Sigma-Aldrich) and 4-methylbellifernoe as a standard (Sigma-Aldrich) [42]. Enzyme activity was inhibited by heating the culture to 100 °C for 2 min and this was used as a zero sample in order to eliminate background levels caused by cyanobacterial photopigments. The methane emission rate was determined by transferring 2 mL of *N. spumigena* UHCC 0039 culture to 12 mL Exetainer^®^ vials with Double Wadded Exetainer^®^ Cap (Labco) in 47 replicates, which were incubated for 0–32 h. In total, 2 mL samples of *N. spumigena* UHCC 0039 and UHCC 0060 cultures were also transferred to 12 mL Exetainer^®^ vials with Double Wadded Exetainer^®^ Cap and incubated for 24 h using the original cultivation conditions to measure the methane emission in different stages of the experiment. Methane release to gaseous environment was analyzed by the headspace technique as follows. Gas samples of 8 mL (at atmospheric pressure) were taken from the headspace with a polypropylene syringe attached to a hypodermic needle and injected into helium-flushed and pre-evacuated 3 mL vials. Gas concentrations (cm^3^ m^−3^) were analyzed using an Agilent GC 7890 custom gas chromatograph equipped with thermal conductivity, flame ionization, and electron capture detectors [43].

### Reverse transcriptase-quantitative PCR (RT-qPCR) and RNA-sequencing

Samples for transcriptomic studies were collected at days 0, 12, and 24 from the cultures containing no phosphorus, P_i_, MPn, and 2APn. RNA samples were fixed with a solution, containing 10 % ethanol and 5 % of phenol and filtered through a 0.22 µm pore diameter polycarbonate filters (GE Water and Process Technologies). RNA was isolated from the filters using RNeasy mini kit (Qiagen) and genomic DNA was degraded using the TURBO DNA-free™ kit (Life Technologies). Ribosomal RNA was removed using MICROBExpress™ Bacterial mRNA enrichment kit (Life Technologies) and complementary DNA (cDNA) libraries were prepared using Bacterial ScriptSeq Complete Kit (Illumina). Total transcriptomes of *N. spumigena* UHCC 0039 in control (P_i_) and MPn treatment on day 12 were sequenced in three replicates of control and in two replicates of treatment, at the Institute for Molecular Medicine Finland (FIMM). Paired-end Illumina HiSeq 2500 RNA-sequencing data were deposited under the accession number of PRJNA388731 and downstream data analysis was performed as described in supplementary information.

*N. spumigena-*specific primers were designed to amplify *phnD*, *phnJ*, *pstS*, and *gyrB* target genes (Table 1). The RT-qPCR analysis was performed using a CFX96 qPCR device (Bio-Rad) and analyzed with the CFX Manager (Bio-Rad). The RT-qPCR reaction mix consisted of 10 ng template cDNA, 300 nM of both primers (Oligomer), and 10 µl of Power Up^TM^ SYBR Green Master Mix (Thermo Fisher Scientific) in a total volume of 20 µL. Purified water was used as a negative control. The qPCR cycling parameters used were as follows: 95 °C for 7 min, 40 × (95 °C for 10 s, 60.5 °C for 30 s) and 95 °C for 10 s. The annealing temperature for melting curve analysis was set from 65 to 95 °C for 5 s to determine amplification of specific product. The amplification efficiency for each primer pair was calculated from the regression slope of standard curve (Figure S1). The relative gene expression was determined by ddCT method comparing values between *gyrB* and target genes *phnJ*, *pstS*, and *phnD* in P_i_, -P_i_ and treatments. Three technical replicates were used.

## Results

### Distribution of *phn* gene cluster

**Fig. 1:**
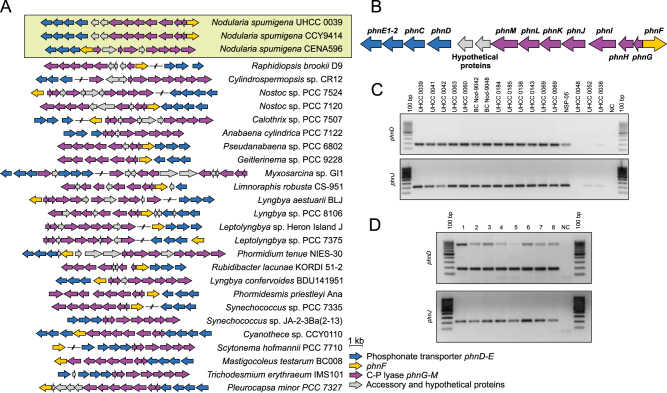
Schematic presentation of *phn* gene clusters found from 28 sequenced cyanobacterial strains **a**. Annotated *phn* gene cluster of *Nodularia spumigena* UHCC 0039 located at 535027–547520 bp **b**. Screening for *phnD* (upper panel) and *phnJ* (lower panel) genes from the *Nodularia* sp. cyanobacteria isolated from the Baltic Sea **c** and environmental samples from the Baltic Sea **d**. Detailed description of *Nodularia* sp. strains and environmental sampling points can be found from Tables S2 and S3

Bioinformatics analysis demonstrates that the phosphonate gene cluster is widely distributed in the cyanobacterial phylum (Fig. 1). A total of 27 out of 500 (5.4%) sequenced cyanobacterial genomes in the NCBI database were found to encode complete or nearly complete sets of genes for phosphonate transport (*phnC-E*) and the C-P lyase complex (*phnG-M*) (Fig. 1a). The *phn* gene clusters lacked clear synteny and the size of the *phn* gene clusters ranged from 8 to 21 kb with the size variation due to gene deletions, duplications, and accessory/hypothetical proteins located between core genes (Table S2). All three strains of *N. spumigena*, for which genome sequences are available, encoded the *phn* gene cluster (Fig. 1a). The *phnJ* and *phnD* genes were also common in *N. spumigena* strains but were absent from the other species of the *Nodularia* genus tested and from *Aphanizomenon flos-aquae* (Fig. 1b and Table S2). We amplified *phnD* and *phnJ* genes from the environmental samples collected from the Baltic Sea using the *N. spumigena*-specific *phnD* and *phnJ* primers demonstrating that these genes are abundant in the Gulf of Finland and the Baltic Proper (Fig. 1c).

### Phosphonates as a sole source of phosphorus

**Fig. 2:**
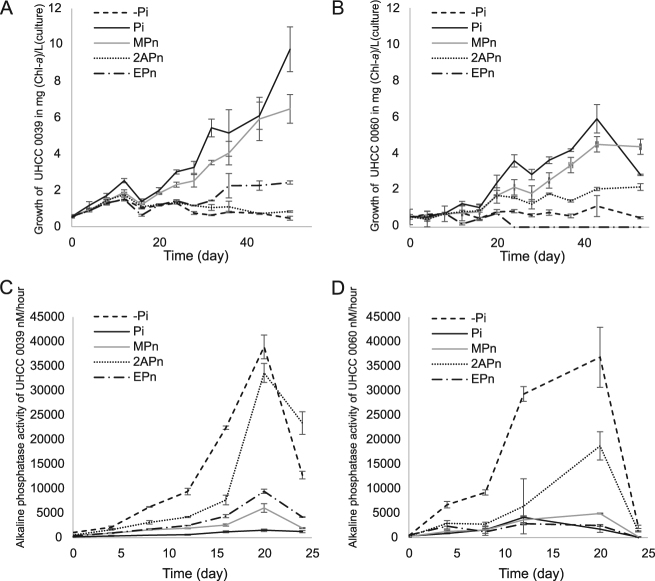
Growth **a**,** b** and alkaline phosphatase activity **c**,** d** of *N. spumigena* UHCC 0039 (left panel) and UHCC 0060 (right panel) in the presence of phosphonates. -P_i_ no phosphorus, P phosphate, MPn methylphosphonate, 2APn 2-aimonmethylphosphonate, EPn ethylphosphonate

*N. spumigena* UHCC 0039 and UHCC 0060 were both able to grow in medium containing MPn (Figs. 2a, b). Minor growth was observed when *N. spumigena* UHCC 0039 grew in the presence of EPn and *N. spumigena* UHCC 0060 in the medium containing 2APn. None of the tested strains were able to utilize the anthropogenic phosphonates etidronic acid monohydrate, *N*-(phophonomethyl) glycine, nitrilotri(methylphosphonic acid), or 2-phosphonobutane-1,2,4-tricarboxylic acid tested here. Maximal growth rates in studied conditions were obtained in the cultures growing in P_i_ medium. Moreover, minor growth in -P_i_ medium was measured indicating that they had not exhausted internal polyphoshphate stores (Figs. 2a, b).

Alkaline phosphatase activity was followed during the cultivation experiment. Alkaline phosphatase activity peaked in both strains in the negative control, when phosphorus was omitted from the medium (Figs. 2c, d). Remarkably elevated alkaline phosphatase activity was additionally observed in 2APn cultures but enzyme activity was reduced compared with -P_i_ medium (Figs. 2c, d). Elevated alkaline phosphatase activity was not observed in MPn supplemented medium despite the absence of P_i_ from the growth medium (Figs. 2c, d).

**Fig. 3:**
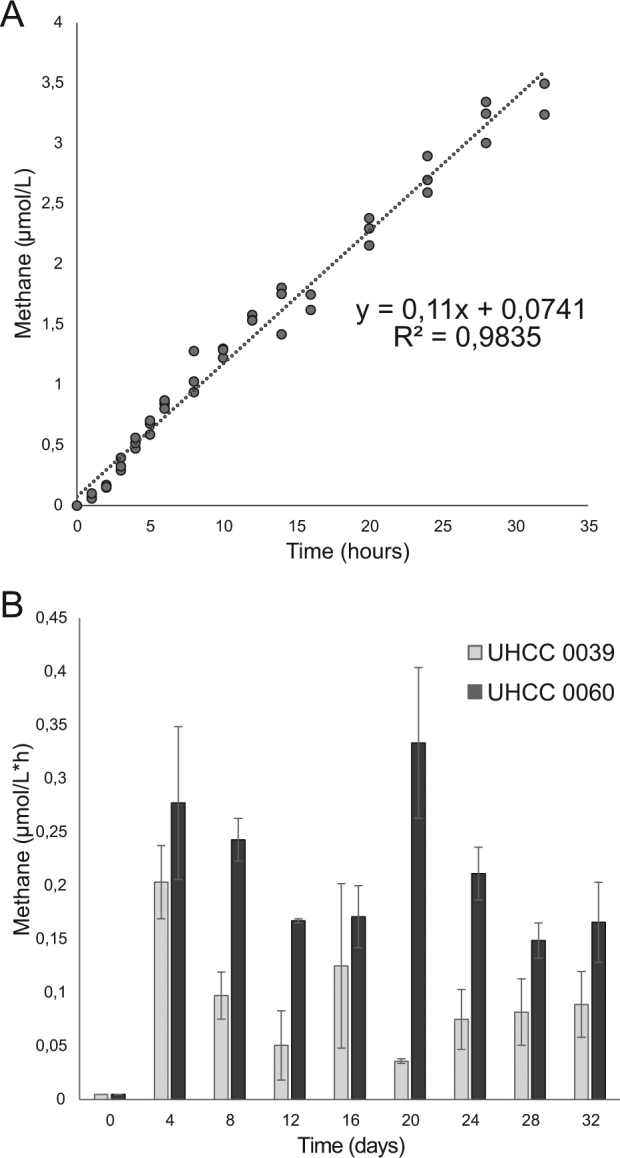
Liberation of methane by *N. spumigena* UHCC 0039 in the medium containing MPn during 32 h **a** and amount of the methane release by *N. spumigena* UHCC 0039 and UHCC 0060 during the growth experiment **b**. Based on the slope of cumulative methane release measurement **a** average methane release was determined to be 1.84 nmol h^−1^ per mg of chlorophyll *a* while chlorophyll *a* concentration was 2.4 mg L^−1^ in the culture

Degradation of phosphonates by the C-P lyase complex releases not only phosphate for the use of the cells but also the organic byproduct, e.g. methane. The rate of the methane flux from *N. spumigena* UHCC 0039 culture to the gaseous environment was determined to be 1.84 nmol h^−1^ per mg of chlorophyll *a*, while prevailing chlorophyll *a* concentration in the culture was 2.4 mg L^−1^ (Fig. 3a). Release of methane was observed in cultures of both strains (Fig. 3b) but emission rate differed remarkably in different growth phases. Overall, *N. spumigena* UHCC 0060 seemed to release more methane compared *N. spumigena* UHCC 0039.

### Transcriptional remodeling

**Fig. 4:**
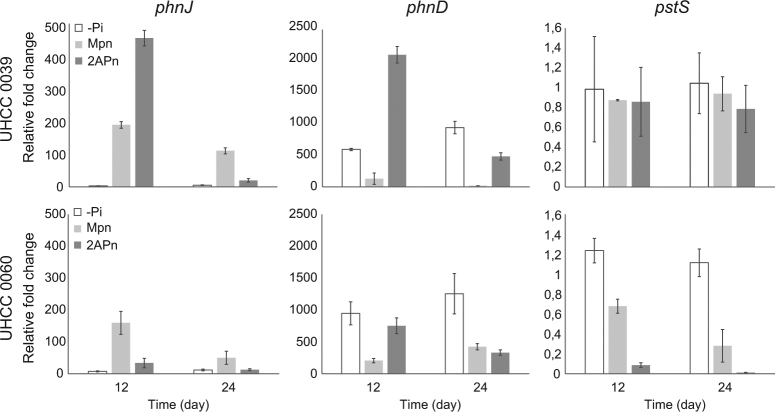
Relative abundance of *phnJ*, *phnD* and *pstS* transcripts based on RT-qPCR analysis in *N. spumigena* UHCC 0039 (upper panel) and UHCC 0060 (lower panel) compared with P_i_ condition. -P_i_ no phosphorus, MPn methylphosphonate, 2APn 2-aimonmethylphosphonate. Note the different scales

MPn and 2APn conditions were selected to analyze the expression levels of phosphorus utilizing genes, compared against the phosphorus-replete condition (P_i_), with phosphorus-deplete condition (-P_i_) as the negative control. Specifically, the gene expression levels of *phnD*, *phnJ*, and *pstS* were investigated in *N. spumigena* UHCC 0039 and UHCC 0060 using RT-qPCR. Although the C-P lyase complex is thought to belong to the *pho* regulon, the expression of *phnJ* was upregulated only in the presence of MPn (Figs. 4). The *phnJ* gene was upregulated on day 12, in both MPn and 2APn conditions, whereas expression remained upregulated only in MPn treatment for 24 days. This provided further evidence that MPn was a suitable phosphorus source for the studied Baltic Sea *N. spumigena* cyanobacteria. The *phnD* gene was upregulated in -P_i_ and 2APn conditions (Figs. 4). Upregulation of the *phnD* gene was minor in MPn treatment compared with P_i_ treatments. Gene expression of *pstS* gene was additionally studied due to its suggested suitability as a marker for P_i_ scarcity but in RT-qPCR analysis differences in *pstS* gene expression were not found (Figs. 4).

**Fig. 5:**
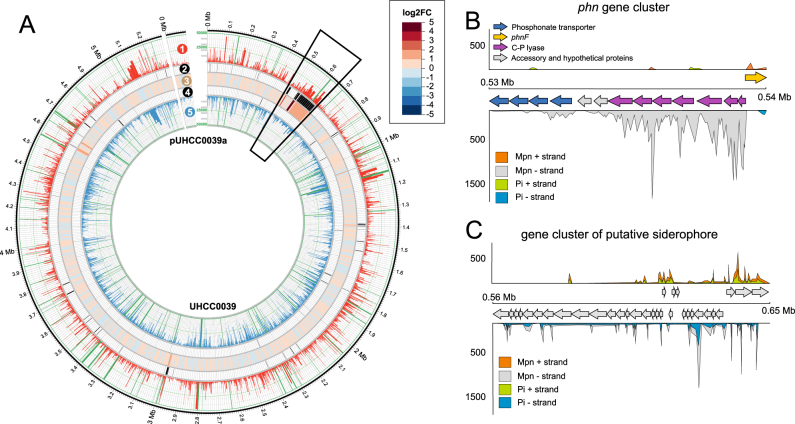
Circular presentation of the *N. spumigena* UHCC 0039 genome along with illustration of RNA-seq data **a** and schematic zoom-in figures of *phn* gene cluster **b** and putative siderophore gene cluster **c**. The rings of the circos plot from outermost to innermost. (1) mean per gene read count of sample grown in MPn condition, (2) upregulated differentially expressed genes, (3) log2FC heat map, (4) downregulated differentially expressed genes, and (5) mean per gene read count of sample P_i_. A maximum read count of 50,000 was set on rings (1) and (5) for visualization purpose. Log2FC = 2 times logarithmic fold change

MPn was the only phosphonate tested that enabled good growth and proper cellular functioning of studied *N. spumigena* strains. We compared the transcriptomes of *N. spumigena* UHCC 0039 grown in MPn and normal P_i_ conditions using RNA-Seq to unravel the transcriptomic responses of phosphonate treatment. In all, 84 upregulated and 8 downregulated genes were determined, by applying the false discovery rate cut-off of <0.01, accounting for a relatively small fraction of all genes (1.8 %). This provided further evidence that MPn permitted normal cellular functioning of *N. spumigena* UHCC 0039 (Fig. 5, Table 2, Table S4). The *phn* gene cluster in *N. spumigena* UHCC 0039 contains 14 genes, of which four (*phnC-E*12) are responsible for phosphonate transport, *phnF* acts as a regulator and *phnG-M* compose a complex cleaving the C-P bond (Fig. 1a). The two additional genes within the *phn* gene cluster of *N. spumigena* UHCC 0039 may be related to accessory or regulatory proteins. The C-P lyase complex (*phnG-M*) was heavily upregulated when phosphonate was present and was the strongest differentially expressed (DE) genomic region (Figs. 5a, b). Significant upregulation was also found for *phnC-E*12 and the two putative regulatory genes, but expression was more inconspicuous. The upstream region of the *phnF* gene was additionally highly expressed in MPn treatment, whereas expression of the coding region of *phnF* was weaker. By contrast, increased expression was found for the antisense strand of *phnF* in the P_i_ control, suggesting an inhibitory role in gene regulation. The genome of *N. spumigena* UHCC 0039 contains also a secondary operon for phosphonate transport (*phnC-E*), which might be involved in the uptake of another forms of phosphorus, for example, organophosphates or phosphites. Transcription and upregulation of *phnC* and *phnD* in this secondary operon indicated that they could be functional (Table 2). Among the upregulated DE genes participating in phosphorus metabolism, high-affinity phosphate transport system *pstABCS* (Log2FC 0.99–2.1) and one atypical alkaline phosphatase *phoA* (BMF81_00431) together with hypothetical protein BMF81_00430, were upregulated (Table 2). Increased expression of *pstABCS* was unexpected, because upregulation of *pstS* was not found in the RT-qPCR study. This is most probably due to the different cDNA library protocols because initial transcriptome was the same and efficiency of the primers was high (Figure S1).

**Table 2:** Selected differentially expressed genes of *Nodularia spumigena* UHCC 0039 while growing in medium containing methylphosphonate as a sole phosphorus source

	Gene name	Function	locus_tag	Log2FC*	*p*-Value
Phosphorus metabolism					
	*pstA*	High-affinity phosphate transporter	BMF81_03296	1.387	5.208e-06
	*pstB*	High-affinity phosphate transporter	BMF81_03295	0.831	4.261e-05
	*pstC*	High-affinity phosphate transporter	BMF81_03297	1.546	2.626e-06
	*pstS*	High-affinity phosphate transporter	BMF81_03298	2.165	6.868e-08
	*phnC2*	Phosphonate ABC-transporter	BMF81_03014	0.907	1.108e-04
	*phnD2*	Phosphonate ABC-transporter	BMF81_03015	0.769	3.924e-05
		Hypothetical protein	BMF81_00430	2.575	1.334e-06
	*phoA*	Alkaline phosphatase	BMF81_00431	3.607	3.972e-07
Photosynthesis					
	*pbsC1*	Chlorophyll a/b binding light-harvesting protein	BMF81_02614	1.542	3.072e-05
	*pbsC2*	Chlorophyll a/b binding light-harvesting protein	BMF81_02616	1.570	1.752e-05
		Flavodoxin, chain A	BMF81_02618	1.642	5.378e-06
		Hypothetical protein	BMF81_02619	1.486	1.715e-05
	*pbsC3*	Chlorophyll a/b binding light-harvesting protein	BMF81_02620	1.768	8.771e-06
	*pbsC4*	Chlorophyll a/b light-harvesting protein	BMF81_02622	1.967	1.106e-06
		Plastocyanin	BMF81_00249	0.996	1.400e-04
Iron uptake					
		Regulatory protein	BMF81_03100	1.222	2.128e-06
		TonB-dependent receptor	BMF81_01155	1.435	1.970e-05
		TonB-dependent receptor	BMF81_01958	0.881	3.238e-05
		ABC-type Fe3+-siderophore transport system permease component	BMF81_01159	1.140	1.782e-05
		ABC-type Fe3+-siderophore transport system permease component	BMF81_01160	1.020	4.858e-05
		ABC-type Fe3+-siderophore transport system permease component	BMF81_01161	1.215	3.983e-06
		Hypothetical protein	BMF81_01162	1.053	2.535e-04

* Log2 fold change

The MPn treatment seemed to co-induce an iron starvation response and upregulated genes related to light-harvesting complex of photosynthesis (Fig. 5, Table 2). The iron stress-induced protein (IsiA) is heavily upregulated under iron-limiting conditions in many cyanobacteria [44, 45] and it is usually used as a marker gene for iron scarcity. The *isiA*/*psbC*/*pcb* genes are clustered together in *N. spumigena* UHCC 0039 and also contain a flavodoxin associated hypothetical protein and a hypothetical protein containing a/b hydrolase domain. Genes belonging to iron starvation and acquisition systems, containing upregulated genes from the *isiA*/*isiB*/*psb* family (BMF81_02614; BMF81_02616; BMF81_02618-20, and BMF81_02622), TonB-dependent receptors (BMF81_01155 and 01958), and Fe^3+^-siderophore transport system permease components (BMF81_01159-61), were induced in MPn treatments (Table 2). In addition, increased expression was found of a genomic region encoding 34 genes (BMF81_00493-00526) (Fig. 5c). The first part of this region includes a non-ribosomal peptide synthetase/polyketide synthase (NRPS/PKS) gene cluster and may encode siderophore. An AraC-type transcriptional regulator (BMF81_03100), which has been suggested to regulate operons involved in iron uptake, was also upregulated.

## Discussion

The availability of P_i_ in the Baltic Sea is scarce during the late summer [3, 34, 46]. Despite limited P_i_ availability, *N. spumigena* forms massive toxic blooms in the Baltic Sea every summer [32, 47]. Alkaline phosphatase is a well-known enzyme that degrades organic phosphoesters [2, 3] (Van Wambeke et al. 2002) and this enzyme enhance the fitness of *N. spumigena* during P_i_ limitation [35]. However, *Nodularia spumigena* CCY 9414, isolated from the Baltic Sea, encodes a *phn* gene cluster indicating it could degrade phosphonates and liberate P_i_ to meet cellular demand [36]. In this study we found *phnD* and *phnJ*, key genes of *phn* gene cluster, from all 12 investigated *N. spumigena* strains, as well as from two other previously sequenced *N. spumigena* CCY 9414 [36] and *N. spumigena* CENA 596 [48] strains showing that this gene cluster is common among *N. spumigena* (Fig. 1), but not in other *Nodularia* spp., *Aphanizomenon flos-aquae* or *Dolichospermum* spp. strains isolated from the Baltic Sea (Fig. 1b, unpublished data). The *phn* gene cluster was additionally found to be ubiquitous in the Baltic Sea using *N. spumigena*-specific primers probing environmental samples (Fig. 1c). Our bioinformatics analysis further showed that only 5.4% of the sequenced cyanobacteria deposited in GenBank carry this particular genetic region and the lack of clear synteny indicates that cyanobacteria have acquired the *phn* gene cluster by horizontal gene transfer. This has also been demonstrated by phylogenetic analyses of *phnJ* gene [26, 49]. The sporadic distribution of the *phn* gene cluster within the cyanobacterial phylum has also been described earlier [50]. This study confirms the hypothesis that *N. spumigena* could degrade phosphonates to cope with the lack of available P_i_ in the Baltic Sea during summer [36] (Nausch, 1998) and may provide *N. spumigena* an important competitive advantage through utilization of phosphonates as an alternative source of phosphorus.

Previous studies have shown that cyanobacteria harboring phosphonate transport and C-P lyase units of the *phn* gene cluster can exploit a broad range of phosphonates such as methyl- and ethylphosphonates, 2-aminoethylphosphonate and glyphosate [27, 28]. The majority of these strains comprise also the regulatory gene *phnF* despite *phnF* is missing from *Trichodesmium erythraeum* IMS101 and *Synechococcus* sp. JA-2-3BA(2–13). However, both strains are nevertheless capable of degrading phosphonates [25, 26]. The small pore size of the PhnC-E channel and intracellular location of C-P lyase complex may be the reasons for limited utilization of phosphonate substrates. Furthermore, variations in amino-acid composition of PhnD proteins affecting the dissociation constants to different phosphonates [51] can also limit the usage of phosphonates. The *phn* gene cluster is thus not sufficient alone to confer the capacity to degrade phosphonates and physiological studies are needed to complement the genomics data to answer the question, which phosphonates are suitable for cyanobacteria.

Here we examined the growth of *N. spumigena* strains UHCC 0039 and UHCC 0060 carrying the *phn* gene cluster in seven different phosphonate supplemented media. MPn fulfilled the phosphorus demand in both strains and 2-aminoehtylphosphonate acted as phosphorus source for *N. spumigena* UHCC 0039 and ethylphosphonic acid to *N. spumigena* UHCC 0060 but two latter substrates enabled only minor growth. Our results suggest that the Baltic Sea *N. spumigena* cyanobacteria can exploit naturally produced phosphonates among which MPn was the preferred form. MPn is suggested to be produced in oceans by heterotrophic microbes expressing a MpnS-dependent biosynthetic pathway [8] and liberation of MPn to the surrounding environment was estimated to be significant due to the short life cycle of these microbes [8]. MPn may thus serve as a phosphorous reservoir for cyanobacteria capable to C-P bond cleavage and this character may explain more intensive occurrence of *N. spumigena* compared with other diazotrophic cyanobacteria when inorganic phosphorus is absent from the upper water layers.

MPn is additionally a precursor for aerobic methane release [29] and circulation of MPn in the oceans is an important contributor in the methane flux to the atmosphere [4]. Based on our results, blooms of *N. spumigena* may also have an important role in aerobic methane release and explain temporal variation of the methane concentration in the Baltic Sea [37, 38]. In addition, phosphonates may increase N_2_-fixation in P_i_-limited environment because N_2_ fixation positively correlates with phosphorus availability [34, 46, 52, 53]. Flux of organic nitrogen to water body [11] further enhance eutrophication in water ecosystems [54]. Finally, nutrient-replete conditions also increase toxin production in *N. spumigena* (Lehtimäki et al. 1997). The anthropogenic phosphonates used in this study were not suitable phosphorus sources for studied *N. spumigena strains* and the riverine load of anthropogenic phosphonate compounds most probably does not enhance *N. spumigena* blooms in the Baltic Sea ([55], this study). However, light intensity used was low compared with conditions *N. spumigena* encounter in the environment [56] although it is likely that *N. spumigena* experience such low light conditions also in nature. Low light conditions resulted in relatively long doubling times of the cultures. In spite of this, studied *N. spumigena* strains were able to grow in MPn medium and release methane.

Genes assisting cyanobacteria to cope with phosphorus-limited conditions belong to the *pho* regulon, which are activated by P_i_ scarcity. One widely used marker for detecting the activation of *pho* regulon and further P_i_ scarcity is alkaline phosphatase activity. In our study, alkaline phosphatase activity decreased in both MPn and 2APn conditions compared with -P_i_ condition. MPn did not induce alkaline phosphatase activity despite the lack of P_i_, indicating an alternative phosphorus uptake pathway might be dominating the process. The *phn* gene cluster belongs to the *pho* regulon [16, 26] and thus should be induced in the lack of P_i_. The C-P lyase part of the *phn* gene cluster was heavily upregulated in the presence of MPn, whereas upregulation of phosphonate transport system was slighter. RT-qPCR studies further validated that *phnD* gene was upregulated in 2APn and -P_i_ control but constant upregulation of *phnJ* gene required suitable phosphonate substrate. Due to the bipartite structure of the *phn* gene cluster, *phnD-E* is most probably regulated independently and the role of *phnF* for the regulation of *phnC-E* may be crucial [24]. In addition, *phnJ* gene was found to be expressed only in the presence of suitable substrate (MPn). Chemical detection of phosphonates in saline water ecosystems is challenging and requires specific analytic tools (nuclear magnetic resonance spetroscopy, NMR) [4]. Thus, transcripts of *phnJ* gene may be suitable indicator for phosphonate bioavailability. Other genes of the *pho* regulon, such as the PstABCS system, were additionally upregulated in MPn condition showing that even though a suitable phosphorus source was available, the *pho* regulon remained activated.

Growth in MPn had little effect on the transcriptome of *N. spumigena* UHCC 0039. However, the treatment resulted in the co-induction of genes related to the light-harvesting complex, as well as iron starvation and acquisition. Expression of the genes encoding photopigments are sensitive to environmental changes and fine tuning the pool of photopigments is an important way for cyanobacteria in adapting to new environment [57]. The iron scarcity marker gene *isiA* becomes usually highly expressed under iron starvation [44]. The *isiA* gene in *N. spumigena* is located together with genes encoding CP43/Pcb family proteins [36] and in our study the whole locus was heavily upregulated in the presence of MPn. Siderophore-mediated iron uptake in cyanobacteria is one mechanism used to overcome iron limitation [58]. However, siderophores are not sufficient for efficient iron scavenging, because these molecules need to be transported to the extracellular space and then the iron-siderophore complex back to the cell [59]. TonB-dependent carriers together with ABC-type transporters are crucial in iron transport. In our study, MPn treatment induced expression of TonB-dependent receptors and Fe-siderophore transporters, as well as an AraC-type regulator, which has a role in the regulation of gene expression. No known siderophores were identified from the genome of *N. spumigena* UHCC 0039. However, the strain carries a genetic region similar to *Nostoc* sp. PCC 7120, which has an important role in iron metabolism [60]. This particular gene cluster has also a great similarity to a siderophore-coding cluster in *Agrobacterium tumefaciensis* [61]. The gene cluster of this putative siderophore in *N. spumigena* UHCC 0039 was heavily upregulated in the MPn condition, but the product remained elusive in this study. Iron constitute cluster with sulfur in the active site of PhnJ [62] and enhanced production of PhnJ may thus cause demand of iron in the cells. In addition, phosphorus and iron are the major nutrients limiting the growth of diazotrophic cyanobacterial blooms during the summer in the Baltic Sea and co-regulation of the genes suggests a hardwired strategy to deal with nutrient limitation in *N. spumigena*.

## Conclusion

Here we demonstrate that strains of *N. spumigena* can grow using methylphosphonate as a sole source of phosphorous with only minor remodeling of the transcriptome. Our results show that *phn* gene clusters are wide-spread in the genomes of the toxic bloom-forming *N. spumigena*. This particular genetic element may enable *N. spumigena* to cope with P_i_-limited conditions by degrading phosphonates and liberating phosphate for the cellular usage. Alkaline phosphatase activity, a marker used to indicate P_i_ starvation, was not detected from the culture growing in the medium supplemented by MPn but a transcriptional response of *pstS*, the other molecular marker used for P_i_ scarcity, was found. *N. spumigena* may contribute to methane supersaturation in the water column by degrading MPn to release phosphate and methane.

## Electronic supplementary material

Supplementary information

Supplementary table S1

Supplementary table S2

Supplementary table S3

Supplementary table S4

Figure S1
