# Supplementary material for: Strains of the toxic and bloom-forming Nodularia spumigena (cyanobacteria) can degrade methylphosphonate and release methane
Source: ISME J. 2018 Feb 14;12(6):1619–30. doi: 10.1038/s41396-018-0056-6 (PMC5955973; doi:10.1038/s41396-018-0056-6)
Supplement: Supplementary file 1 — Supplementary information [file 41396_2018_56_MOESM1_ESM.docx]

**Supplementary information**

*RNA-seq data analysis*

Original paired-end library sizes for RNA-seq data were 55806285, 57997441 and 58613917 for control and 68974269 and 50334330 for MPn treatment. FastQC v0.11.4 was used to check the quality of the raw reads (Andrews, 2010), and reads that passed the quality control were demultiplexed to 101 bp using Trimmomatic with phred score 33. Clean reads were aligned to reference genome of *N. spumignea* UHCC 0039 using BWA-MEM v0.7.7 (Li, 2013) in paired-end mode with default parameters. Aligned SAM format reads were converted to BAM format applying SAMtools v1.2 (Li *et al*., 2009). Reads were further filtered with BAM filter (Galaxy Version 0.5.7.1) to remove unmapped reads, reads less than 20 nt long, flagged as secondary alignments or marked as PCR duplicates as well as reads with low quality. SAMtools v1.19 (Li *et al*., 2009) was used to sort filtered BAM files along the chromosomal coordinates. Unambiguously aligned fragments to genomic features were quantified by FeatureCounts v1.4.6.p5 (Liao *et al*., 2014) considering only paired-end reads constitutin a fragment size of 50 ~ 600 nt. Moreover, chimeric fragments were excluded. RNAsequencing analysis was run using the University of Freiburg Galaxy interface (Afgan *et al*., 2016; Cock *et al*., 2013).

Prior to calling the differentially expressed genes, weakly expressed features were removed. First, the counts were converted to Count Per Million (CPM) and features passing the criteria, sum of CPM across all samples should be more than 3 and at least 3 samples had a CPM more than 1, were taken for further analysis. In addition rRNA and tRNA were excluded. The differentially expressed genes were identified using edgeR (Robinson *et al*., 2010) and DESeq (Anders and Huber, 2010) following the earlier described protocol (Anders *et al*., 2013) and differentially expressed genes were illustrated using Circos (Krzywinski *et al*., 2009*).*

Furthermore, to illustrate RNAseq data in artemis, BAM files were converted to Wiggle format using the paired end mode of bam2wig.py from the RseQC v2.4 package (Wang *et al*., 2012). and wiggle format was normalized to 1000000000 wigsum. Customized script (<https://github.com/housw/GRPutils>) was used to provide Artemis compatible genome coverage graphs (Carver *et al*., 2012).

*References*

Afgan E, Baker D, van den Beek M, Blankenberg D, Bouvier D, Čech M, *et al*. (2016). The Galaxy platform for accessible, reproducible and collaborative biomedical analyses: 2016 update. *Nucleic Acids Res* **44**: W3–10.

Anders S. Huber W. (2010). Differential expression analysis for sequence count data. *Genome Biology* **11**: R106.

Anders S, McCarthy DJ, Chen Y, Okoniewski M, Smyth GK, Huber W, *et al*. (2013). Count-based differential expression analysis of RNA sequencing data using R and Bioconductor. *Nat Protocols* **8**: 1765–1786.

Andrews S. (2010). FastQC: A Quality Control tool for High Throughput Sequence Data. Available Online at http://www.bioinformatics.babraham.ac.uk/projects/fastqc/.

Carver T, Harris SR, Berriman M, Parkhill J, McQuillan JA. (2012). Artemis: an integrated platform for visualization and analysis of high-throughput sequence-based experimental data. *Bioinformatics* **28**: 464–469.

Cock PJA, Grüning BA, Paszkiewicz K, Pritchard L. (2013). Galaxy tools and workflows for sequence analysis with applications in molecular plant pathology. *PeerJ* 1: e167.

Krzywinski M, Schein J, Birol I, Connors J, Gascoyne R, Horsman D, *et al*. (2009). Circos: an information aesthetic for comparative genomics. *Genome Res* **19**: 1639–1645.

Li H. (2013). Aligning sequence reads, clone sequences and assembly contigs with BWA-MEM. Retrieved from http://arxiv.org/abs/1303.3997

Li H, Handsaker B, Wysoker A, Fennell T, Ruan J, Homer N, *et al*. (2009). The Sequence alignment/map format and SAMtools. *Bioinformatics* **25**: 2078–9.

Liao Y, Smyth G K, Shi W. (2014). FeatureCounts: An efficient general purpose program for assigning sequence reads to genomic features. *Bioinformatics* **30**: 923–930.

Robinson MD, McCarthy DJ, Smyth GK. (2010). edgeR: a Bioconductor package for differential expression analysis of digital gene expression data. *Bioinformatics* **26**: 139–140.

Wang L, Wang S, Li W. (2012). RSeQC: quality control of RNA-seq experiments. *Bioinformatics* **28**: 2184–5.
