# Supplementary material for: Strains of the toxic and bloom-forming Nodularia spumigena (cyanobacteria) can degrade methylphosphonate and release methane
Source: ISME J. 2018 Feb 14;12(6):1619–30. doi: 10.1038/s41396-018-0056-6 (PMC5955973; doi:10.1038/s41396-018-0056-6)
Supplement: Supplementary file 6 — Figure S1 [file 41396_2018_56_MOESM6_ESM.docx]

**Supplementary figures**

A

B

C

D

**Figure S1** Efficiency of the used primer sets. (a) *phnD*, (b) *phnJ* and (c) *pstS* and (d) *gyrB*.
